# Optimal bowel resection margin in colon cancer surgery: prospective multicentre cohort study with lymph node and feeding artery mapping

**DOI:** 10.1016/j.lanwpc.2022.100680

**Published:** 2023-01-18

**Authors:** Hideki Ueno, Kazuo Hase, Akio Shiomi, Manabu Shiozawa, Masaaki Ito, Toshihiko Sato, Yojiro Hashiguchi, Takaya Kusumi, Yusuke Kinugasa, Hideyuki Ike, Kenji Matsuda, Kazutaka Yamada, Koji Komori, Keiichi Takahashi, Yukihide Kanemitsu, Heita Ozawa, Masayuki Ohue, Tadahiko Masaki, Yasumasa Takii, Atsushi Ishibe, Jun Watanabe, Yuji Toiyama, Hiromichi Sonoda, Keiji Koda, Yoshito Akagi, Michio Itabashi, Takahiro Nakamura, Kenichi Sugihara

**Affiliations:** a Department of Surgery, National Defense Medical College, Saitama, Japan; b Division of Colorectal Surgery, Shizuoka Cancer Center Hospital, Shizuoka, Japan; c Department of Gastrointestinal Surgery, Kanagawa Cancer Center, Kanagawa, Japan; d Colorectal and Pelvic Surgery Division, Department of Surgical Oncology, National Cancer Center Hospital East, Chiba, Japan; e Department of Surgery, Yamagata Prefectural Central Hospital, Yamagata, Japan; f Department of Surgery, Teikyo University School of Medicine, Tokyo, Japan; g Department of Surgery, Keiyukai Sappro Hospital, Hokkaido, Japan; h Department of Gastrointestinal Surgery, Tokyo Medical and Dental University, Tokyo, Japan; i Department of Surgery, Saisei-kai Yokohama-shi Nanbu Hospital, Kanagawa, Japan; j Second Department of Surgery, School of Medicine, Wakayama Medical University, Wakayama, Japan; k Department of Gastroenterological Surgery, Coloproctology Center, Takano Hospital, Kumamoto, Japan; l Department of Gastroenterological Surgery, Aichi Cancer Center Hospital, Aichi, Japan; m Department of Surgery, Tokyo Metropolitan Cancer and Infectious Diseases Center Komagome Hospital, Tokyo, Japan; n Department of Colorectal Surgery, National Cancer Centre Hospital, Tokyo, Japan; o Department of Surgery, Tochigi Cancer Centre, Utsunomiya, Japan; p Department of Gastroenterological Surgery, Osaka International Cancer Institute, Osaka, Japan; q Department of Surgery, Kyorin University School of Medicine, Tokyo, Japan; r Department of Surgery, Niigata Cancer Centre Hospital, Niigata, Japan; s Department of Gastroenterological Surgery, Yokohama City University Graduate School of Medicine, Kanagawa, Japan; t Department of Surgery, Gastroenterological Centre, Yokohama City University Medical Centre, Kanagawa, Japan; u Department of Gastrointestinal and Pediatric Surgery, Mie University Graduate School of Medicine, Mie, Japan; v Department of Surgery, Shiga University of Medical Science, Shiga, Japan; w Department of Surgery, Teikyo University Chiba Medical Centre, Chiba, Japan; x Department of Surgery, Kurume University School of Medicine, Fukuoka, Japan; y Department of Surgery, Institute of Gastroenterology, Tokyo Women's Medical University, Tokyo, Japan; z Laboratory for Mathematics, National Defense Medical College, Saitama, Japan; aa Tokyo Medical and Dental University, Tokyo, Japan

**Keywords:** Colon cancer, Pericolic lymph node, Regional lymph node, Tumour-node-metastasis classification

## Abstract

**Background:**

There are no standardised criteria for the ‘regional’ pericolic node in colon cancer, which represents a major cause of the international uncertainty regarding the optimal bowel resection margin. This study aimed to determine ‘regional’ pericolic nodes based on prospective lymph node (LN) mapping.

**Methods:**

According to preplanned *in vivo* measurements of the bowel, the anatomical distributions of the feeding artery and LNs were determined in 2996 stages I–III colon cancer patients who underwent colectomy with resection margin >10 cm at 25 institutions in Japan.

**Findings:**

The mean number of retrieved pericolic nodes was 20.9 (standard deviation, 10.8) per patient. In all patients except seven (0.2%), the primary feeding artery was distributed within 10 cm of the primary tumour. The metastatic pericolic node most distant from the primary tumour was within 3 cm in 837 patients, 3–5 cm in 130 patients, 5–7 cm in 39 patients and 7–10 cm in 34 patients. Only four patients (0.1%) had pericolic lymphatic spread beyond 10 cm; all of whom had T3/4 tumours accompanying extensive mesenteric lymphatic spread. The location of metastatic pericolic node did not differ by the feeding artery's distribution. Postoperatively, none of the 2996 patients developed recurrence in the remaining pericolic nodes.

**Interpretation:**

The pericolic nodes designated as ‘regional’ were those located within 10 cm of the primary tumours, which should be fully considered when determining the bowel resection margin, even in the era of complete mesocolic excision.

**Funding:**

Japanese Society for Cancer of the Colon and Rectum.

**Research in context**

**Evidence before this study**

Internationally, there is a great deal of uncertainty regarding the optimal resection margin of the bowel in colon cancer surgery. A major reason for this is that there are no standardised criteria for regional pericolic nodes to be resected in colon cancer surgery, and, as per the tumour–node–metastasis classification, all pericolic nodes are treated as ‘regional’. The conventional concept of feeding artery-oriented bowel resection often influences surgeons' readiness to include two or more feeding arteries in the resection specimens. In addition, as a result of the recent prevalence toward performing complete mesocolic excision (CME), the length of the resected bowel is often used as a yardstick for evaluating specimen quality, and wider resection of the bowel is regarded as a hallmark of high-quality colon cancer surgery. However, there is currently no robust evidence for the advantage of wider resection of the bowel.

**Added value of this study**

This is the first large-scale, prospective multicentre cohort study to identify and assess metastatic pericolic lymph node distribution in patients with stage I–III colon cancer. The satisfactory number of pericolic lymph nodes examined and the methodology whereby intraoperative markings were used to identify the location of the lymph nodes and feeding arteries ensured high-quality lymph node mapping. Pericolic lymphatic spread beyond 10 cm was rare (approximately 0.1%). All exceptional cases with positive distant pericolic nodes had T3/T4 tumours with massive lymph node metastasis and experienced a high incidence of recurrence; this finding indicates that such distant pericolic spread is a form of systemic, rather than local disease. Furthermore, in 99.8% of patients, one or more feeding arteries were located in the pericolic region within 10 cm of the primary tumour. Moreover, the distribution of the feeding artery did not significantly affect the status of the pericolic lymphatic spread, although it did affect the mesenteric lymphatic spread toward the origin of the feeding artery. Finally, intraoperative identification of the feeding artery was 95% accurate, but this was reduced to 86% in patients with a BMI ≥30.

**Implications of all the available evidence**

Based on the actual anatomical distribution of pericolic lymph node metastasis and its prognostic implication, the 10 cm-rule is a valid and practical criterion to define regional pericolic nodes. Even in the era of CME, it should be emphasized that surgical procedures intended to extend bowel resection beyond the regional pericolic area do not improve the quality of surgery. This should be fully considered when determining the bowel resection margin in curatively-intended surgery for colon cancer.

## Introduction

Lymph node (LN) metastasis is the most common mode of spread in colon cancer, and the oncological outcomes of patients who undergo curative colectomy are greatly influenced by the quality of surgery, specifically that of lymphadenectomy. In most patients, the pericolic nodes, i.e., the LNs located along the bowel and marginal artery, are the starting site of metastasis.^1^, ^2^, ^3^ The optimal extent of bowel resection is closely associated with how ‘regional’ pericolic nodes are defined, which should be routinely resected due to the potential risk of metastasis. However, there are currently no global standardised criteria for the definition of regional LNs in the pericolic region. As per the tumour-node-metastasis classification, all pericolic nodes are treated as regional, and there are no specific sub-classifications based on the risk of metastasis in individual LNs.^4^ This lack of classification has greatly contributed to the uncertainty regarding the optimal resection margin of the bowel in colon cancer.

For example, in surgical textbooks published in the United States, ‘left partial colectomy’ for tumours located at the descending colon has been introduced as a procedure to resect the transverse colon and sigmoid colon up to the middle of each, as well as all of the sigmoid arteries except for the most distal.^5^ According to this procedure, the resection margin of the colon should reach approximately 20 cm (or longer in some cases) on each side of the primary tumour, although the exact value has not been specified. Meanwhile, the American Society of Colon and Rectal Surgeons (ASCRS) recommends that colon resection includes proximal and distal margins of 5–7 cm in order to ensure the adequate removal of pericolic nodes at risk of metastasis.^6^ In addition, a previous study highlighted a significant international difference in the length of bowel resected in colon cancer surgery. For instance, in a leading German institution adopting complete mesocolic excision (CME), the median length of large bowel specimens resected for left-side tumours was 38 (32–43) cm, but was only 15 (13–20) cm in Japanese institutions adopting D3 dissection.^7^

This multicentre cohort study conducted by the Japanese Society for Cancer of the Colon and Rectum (JSCCR) prospectively assessed metastatic LN distribution, focusing on the distance from the primary tumour and the anatomical association with feeding artery location (UMIN000030331). The main purpose of this study was to determine the criteria for ‘regional’ pericolic nodes to facilitate the standardisation of colon cancer surgery by ascertaining the optimal length of bowel resection.

## Patients and methods

### Patients

This study was conducted according to the Declaration of Helsinki and comparable Japanese ethical standards. The study was approved by the Institutional Review Boards of the JSCCR and each participating institution. Written informed consent was obtained from all study participants.

This is a multicentre, prospective cohort study. The pre-determined target sample size of this study determined on the basis of the consensus of opinion among the investigators in terms of the validity of sub-population analysis according to T stage was 3000, and a total of 3102 patients were enrolled in this study to examine the anatomical distribution of metastatic LNs (Supplemental Fig. S1). After excluding 106 patients who were ineligible for analysis {48 patients with rectal cancer, 14 patients with distant metastasis, one patient with benign disease, 38 patients with missing data, and 5 patients withdrawn from the study based on the request from the institution due to any ineligible criteria which had been found after registration}, 2996 patients with histologically proven adenocarcinoma, classified as pathological stage I, II or III colon cancer, who received curative surgery at 25 institutions in the JSCCR (Supplemental Table S1) between June 2013 and December 2017 and were in principle followed up according to the JSCCR Guidelines^8^ were included. Patients with multiple colon cancers and those treated with preoperative adjuvant therapy were excluded. The patient demographics are shown in Table 1. Because tumour location is an essential factor that can affect the distribution of positive lymph nodes, it was estimated not only with the conventional categorisation (cecum, ascending colon, transverse colon, descending colon, and sigmoid colon: categorisation 1), but with the categorisation of whether the tumor is located at the flexure portion or non-flexure portion of the colon (categorization 2).

**Table 1:** Background of the 2996 colon cancer patients analysed.

Factors	
Age of patients, mean (SD)	68.8 (11.2)
Sex, number (%)	Male, 1528 (51.0) Female, 1468 (49.0)
Body mass index, mean (SD)	22.6 (3.5)
Surgical approach, number (%)	Open, 889 (29.7) Laparoscopic, 2107 (70.3)
Intraoperative marking, number (%)	Under direct vision, 2903 (96.9) Laparoscopically, 93 (3.1)
Number of LN retrieved, mean (SD)	All: 30.8 (15.0) Pericolic: 20.9 (10.8) Intermediate: 6.3 (4.8) Main: 3.6 (4.1)
Main LN dissection, number (%)	Yes: 2436 (81.3) No: 560 (18.7)
Pathological T stage, number (%)	T1: 574 (19.2) T2: 440 (14.7) T3: 1443 (48.2) T4a: 449 (15.0) T4b: 90 (3.0)
Pathological N stage, number (%)	N0: 1880 (62.8) N1: 792 (26.4) N2: 324 (10.8)
Lymph node positivity (%)	Overall: 1116 (37.3) Pericolic: 1044 (34.8) Intermediate: 257 (8.6) Main: 68 (2.8)^a^
Tumour differentiation^b^, number (%)	Well: 1248 (41.7) Mod: 1552 (51.8) Por: 91 (3.0) Muc: 99 (3.3) Others: 6 (0.2)
Tumour location [categorization 1]^c^, number (%)	C: 368 (12.3) A: 989 (33.0) T: 459 (15.3) D: 215 (7.2) S: 965 (32.2)
Tumour location [categorization 2], number (%)	Hepatic flexure: 262 (8.7) Splenic flexure: 154 (5.1) Others: 2580 (86.1)
Proximal and distal length of bowel resected (cm) according to tumour location-1, mean (SD)	C: 11.7 (4.5) and 12.2 (4.4) A: 13.1 (5.5) and 12.2 (4.3) T: 15.7 (7.8) and 11.6 (3.6) D: 11.4 (4.8) and 12.2 (4.9) S: 10.7 (2.7) and 11.4 (3.7)
Proximal and distal length of the resected bowel (cm) according to tumour location [categorisation 2], mean (SD)	Hepatic flexure: 18.1 (8.4) and 11.4 (2.8) Splenic flexure: 11.5 (4.3) and 12.6 (5.5) Others: 11.9 (4.7) and 11.8 (4.1)
Postoperative adjuvant chemotherapy^d^, number (%)	CAPOX: 387 (12.9) [249 (8.3)] Capecitabine: 322 (10.7) [250 (8.3)] UFT/leucovorin: 173 (5.8) [136 (4.5)] FOLFOX: 53 (1.8) [38 (1.3)] SOX: 21 (0.7) [15 (0.5)] S-1: 15 (0.5) [11 (0.4)] 5-fluorouracil/leucovorin: 11 (0.4) [11 (0.4)] none: 2008 (67.0) uncertain: 6 (0.2)

LN: lymph node, SD: standard deviation.

a In patients who underwent main LN dissection (n = 2436).

b Wel, Mod, Por, and Muc represent well differentiated adenocarcinoma, moderately differentiated adenocarcinoma, poorly differentiated adenocarcinoma, and mucinous carcinoma, respectively.

c C, A, T, D, and S represent the cecum, ascending colon, transverse colon, descending colon, and sigmoid colon, respectively.

d CAPOX: a chemotherapy combination including capecitabine and oxaliplatin; UFT: tegafur and uracil; FOLFOX: a chemotherapy combination including 5-fluorouracil, leucovorin, and oxaliplatin; SOX: a chemotherapy combination including S-1 and oxaliplatin; numbers in square brackets represent those of patients who completed adjuvant chemotherapy.

The incidence of recurrence associated with the length of the bowel resected was investigated in these 2996 patients using surveillance information in January 2020. The median follow-up period was 44 (0–76) months. A total of 300 patients (10.0%) developed recurrent disease (local, regional, or distant recurrence), and the 3-year relapse-free survival (RFS) rate was 88.5% as calculated by the Kaplan–Meier method.

### Bowel resection and *in vivo* markings on the bowel and feeding artery

Bowel resection was performed to include all pericolic LNs defined as ‘regional’ according to both the 10-cm rule (Supplemental Fig. S2) and the feeding-artery-oriented rule (Supplemental Fig. S3). Consequently, all patients received colectomy with a bowel resection margin of at least 10 cm. Patients who had no feeding arteries within 10 cm of the primary tumour received a wider bowel resection to include all LNs between the primary tumour and 5 cm from the primary feeding artery.

The anatomical location of the feeding artery and LNs were determined based on preplanned *in vivo* measurements of the bowel. More specifically, before resecting the bowel, in the natural state with no external tension, points were marked respectively on the bowel (the colon or terminal ileum) by serosal sutures at 3, 5, 7 and 10 cm from the proximal and distal edges of the tumour. The distal end of the primary feeding artery was also marked with a stitch during the operation.

### Back-table procedures: Identification of feeding arteries in the resected specimens

**Fig. 1:**
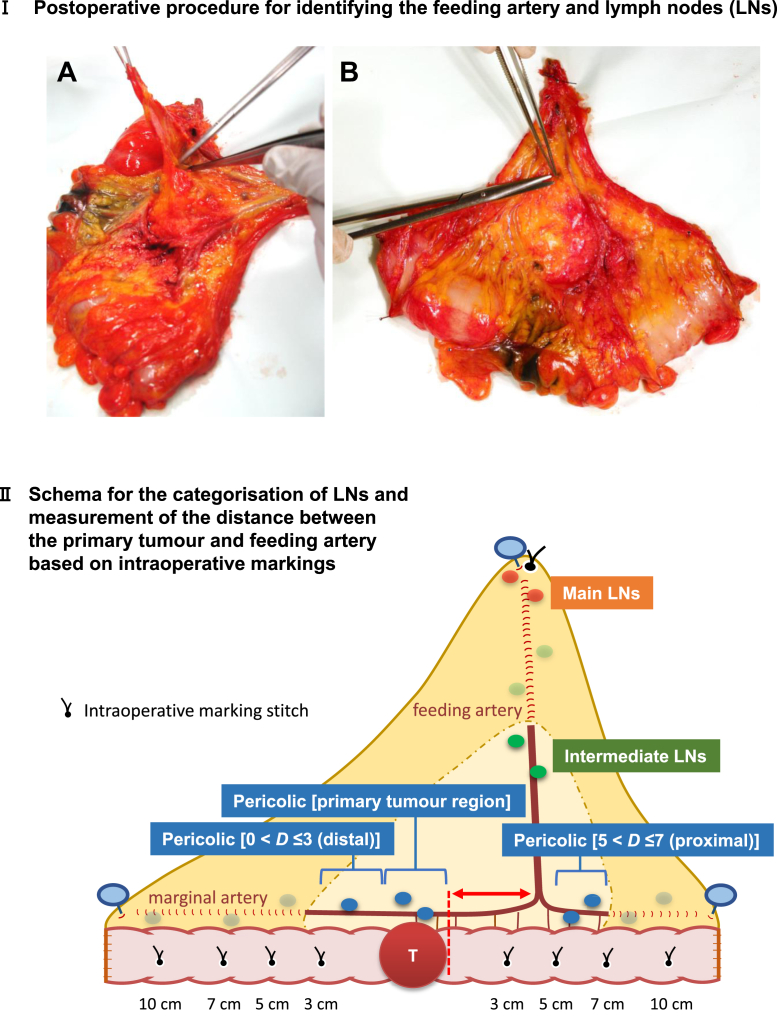
**Identification of the primary feeding artery and lymph nodes, and their categorisation in the resected surgical specimens.** The resected fresh surgical specimens were pinned on corkboard in a fan shape, with the feeding artery stump and both sides of the bowel stumps stretched out, and the mesocolic fascia was peeled off using surgical scissors (IA). The mesenteric fatty tissues were removed piecemeal with scissors to visually identify the feeding/marginal arteries and lymph nodes (IB). The distance between the distal end of the primary feeding artery and the closest edge of the primary tumour (*double-headed arrow*) was recorded according to the intraoperative marking stitches on the bowel (II). The lymph nodes were classified into pericolic, intermediate and main lymph nodes according to the *Japanese Classification of Cancer of the Colon and Rectum* (Supplemental Fig. S4). Pericolic lymph nodes were collected by groups, according to the intraoperative serosal sutures made at 3, 5, 7 and 10 cm from the primary tumour (i.e., primary tumour region, 0 < *D* ≤ 3 [proximal/distal], 3 < *D* ≤ 5 [proximal/distal], 5 < *D* ≤ 7 [proximal/distal], 7 < *D* ≤ 10 [proximal/distal] and *D* > 10 [proximal/distal] groups) and were separately submitted to pathological examination. The abovementioned processes were performed by surgeons. *D*: Distance from the closer primary tumour edge (cm).

After resecting the bowel, the primary feeding artery (i.e., the branch of the superior or inferior mesenteric artery located closest to the primary tumour) was identified in fresh surgical specimens and its anatomical location was recorded (Fig. 1). The distance between the distal end of the primary feeding artery and the closest edge of the primary tumour was determined according to the intraoperative marking stitches on the bowel.

### Back-table procedures: LN retrieval and categorisation

LNs were retrieved from the resected surgical specimens and categorised as pericolic, intermediate, or main LNs based on the LN grouping system of the *Japanese Classification of Colorectal Carcinoma* issued by the JSCCR (Supplemental Fig. S4).^9^^,^^10^ The pericolic nodes were subcategorised according to the distance from the primary tumour (*D*) determined by the intraoperative marking stitches on the bowel (Fig. 1) as follows: (1) primary tumour region, (2) 0 < *D* ≤ 3 cm (proximal/distal sides), (3) 3 < *D* ≤ 5 cm (proximal/distal sides), (4) 5 < *D* ≤ 7 cm (proximal/distal sides), (5) 7 < *D* ≤ 10 cm (proximal/distal sides) and (6) *D* > 10 cm (proximal/distal sides).

The procedures that were performed to identify the feeding/marginal arteries and to retrieve LNs were exclusively performed by surgeons. LNs were pathologically examined in routine diagnostic practice at each institution with the conventional method with hematoxylin and eosin staining for a representative section of the LN. Tumor nodules with no pathological evidence of LN structure are considered as LN metastases, irrespective of their size and contour morphology.

### Statistical analyses

The study endpoints were to determine the anatomical distribution of metastatic LNs, and the incidence of recurrence associated with insufficient lengths of bowel resection (https://upload.umin.ac.jp/cgi-open-bin/ctr_e/ctr_view.cgi?recptno=R000034640).

The analysis of variance (ANOVA) and t-test were used for continuous variables and the chi-square test or Fisher's exact test was used for categorical variables to assess differences in the number of LNs retrieved/involved, feeding artery distribution, accuracy of intraoperative identification of the feeding artery and recurrence rates. Test of Spearman's rank correlation coefficient and Cochran-Armitage trend test were used to assess associations between the location of the primary feeding artery and pericolic tumour spread or tumour spread along supplying arteries. The RFS (time from surgery to the first recurrence or death) was estimated using the Kaplan–Meier method. Alive patients, including those who were lost to follow-up, were censored at their last follow-up date. All statistical analyses were performed using R version 3.6.1 (R Foundation, Vienna, Austria) by an experienced mathematical statistician (TN).

### Role of the funding source

This study was supported by the Fund of the Japanese Society for Cancer of the Colon and Rectum (JSCCR). No pharmaceutical company nor other agency were involved in this study and this research received no specific grant funding with no role of the funding source in this work.

## Results

### Pericolic nodal status according to the distance from the primary tumour

**Fig. 2:**
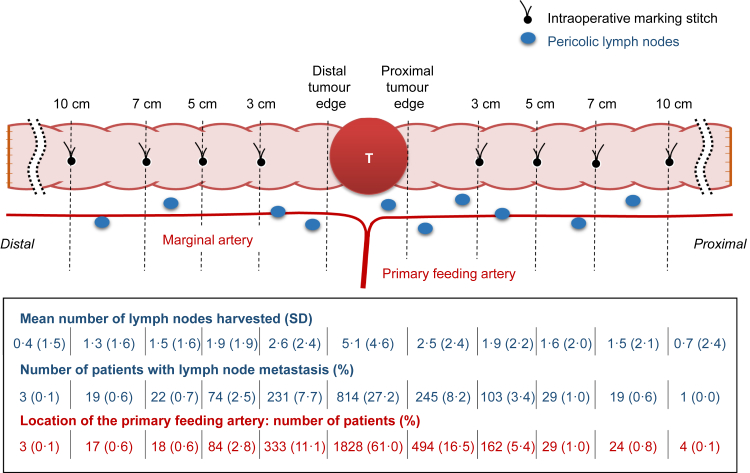
**Actual status of the distribution of pericolic lymph nodes and the primary feeding artery.** The number of lymph nodes, incidence of lymph node metastasis and location of the primary feeding artery are shown according to the segments determined by *in vivo* marking stiches made at 3, 5, 7 and 10 cm from the primary tumour. SD: Standard deviation.

The anatomical distribution of the pericolic LNs is shown in Fig. 2. The primary tumour region had the largest number of LNs {mean, 5.1; standard deviation (SD), 4.6}, and the number decreased according to the distance from the primary tumour. The total number of retrieved pericolic nodes was 20.9 (SD, 10.8) per patient, and the number was significantly associated with the T-stage, ranging from 17.7 (SD, 9.8) for T1 to 25.1 (SD, 10.6) for T4b (Table 2).

**Table 2:** Pericolic lymph nodal status according to pathological T stage.

	T-stage	Overall	*P*-value	Pericolic segments subdivided by the distance from the primary tumour (cm)^a^					
				Primary tumour region	0 < *D* ≤ 3	3 < *D* ≤ 5	5 < *D* ≤ 7	7 < *D* ≤ 10	*D* > 10
Mean and SD of the number of retrieved lymph nodes per patient	T1 (n = 574)	17.7 (9.8)	<0.0001^b^	3.3 (3.4)	4.7 (3.4)	3.6 (2.9)	3.0 (2.8)	2.5 (2.6)	0.8 (2.3)
	T2 (n = 440)	20.7 (10.8)		4.6 (4.3)	5.1 (3.7)	3.9 (3.1)	3.1 (2.9)	2.9 (3.6)	1.0 (2.8)
	T3 (n = 1443)	21.1 (10.1)		5.4 (4.6)	5.1 (3.5)	3.7 (2.9)	3.1 (2.6)	2.7 (2.7)	1.1 (2.6)
	T4a (n = 449)	23.9 (13.0)		6.6 (5.2)	5.4 (3.6)	4.1 (3.1)	3.5 (2.8)	3.0 (2.9)	1.4 (5.3)
	T4b (n = 90)	25.1 (10.6)		7.5 (5.6)	5.8 (3.7)	4.3 (3.1)	3.0 (2.6)	3.2 (3.0)	1.2 (2.8)
	All (n = 2996)	20.9 (10.8)		5.1 (4.6)	5.1 (3.5)	3.8 (3.0)	3.2 (2.7)	2.7 (2.9)	1.1 (3.1)
The number of patients with metastatic pericolic lymph nodes (%)	T1 (n = 574)	68 (11.8)	<0.0001^c^	38 (6.6)	23 (4.0)	15 (2.6)	3 (0.5)	1 (0.2)	0
	T2 (n = 440)	111 (25.2)		75 (17.0)	47 (10.7)	17 (3.9)	5 (1.1)	6 (1.4)	0
	T3 (n = 1443)	534 (37.0)		418 (29.0)	204 (14.1)	83 (5.8)	22 (1.5)	19 (1.3)	2 (0.1)
	T4a (n = 449)	277 (61.7)		235 (52.3)	114 (25.4)	42 (9.4)	16 (3.6)	8 (1.8)	2 (0.4)
	T4b (n = 90)	54 (60.0)		48 (53.3)	18 (20.0)	7 (7.8)	3 (3.3)	1 (1.1)	0
	All (n = 2996)	1044 (34.8)		814 (27.2)	406 (13.6)	164 (5.5)	49 (1.6)	35 (1.2)	4 (0.1)

*D*: distance from the closer primary tumour edge (cm), SD: standard deviation.

a Sum of the number of lymph node (upper row) and patient (lower row) both proximal and distal to the primary tumour.

b ANOVA.

c Chi-square test.

Overall, 1044 (34.8%) out of 2996 patients had pericolic LN metastasis. Among the 11 pericolic segments made according to the *in vivo* marking stiches at 3, 5, 7 and 10 cm from the primary tumour, the incidence of LN metastasis was highest in the primary tumour region (27.2%) and decreased with increasing distance from the primary tumour (Fig. 2). There was no significant difference in the anatomical distribution of the positive pericolic nodes between the proximal and distal sides (*P* = 0.41). The incidence of metastasis in LNs located ≥7 cm from the primary tumour was <1% both proximal and distal to the primary tumour. The incidence according to the T-stage is shown in Table 2.

**Fig. 3:**
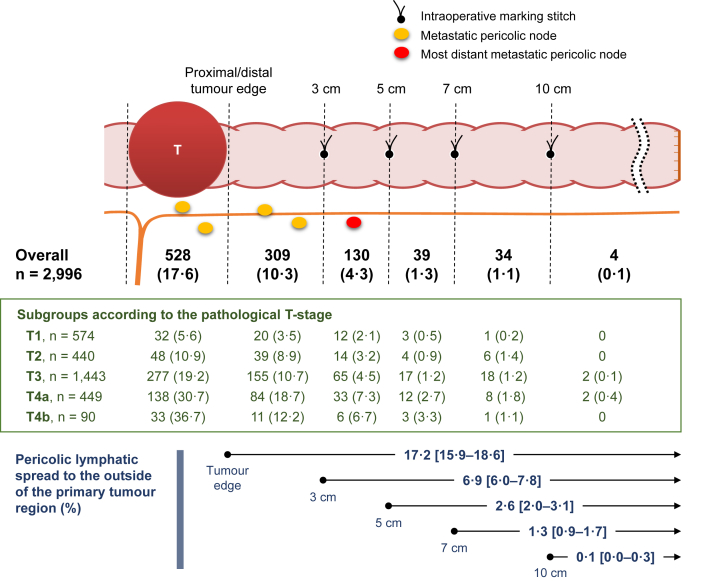
**Location of the most distant metastatic pericolic lymph node.** The pericolic region was divided into six segments according to the *in vivo* marking stiches made at 3, 5, 7 and 10 cm from the primary tumour. The figures demonstrate the number (%) of patients whose most distant metastatic pericolic lymph node was located in each segment, irrespective of being distal and proximal to the primary tumour. Note that the primary tumour region was the most common segment to harbour the most distant pericolic lymph node involvement, and the incidence decreased as the distance from the primary tumour increased. The values in the brackets indicate 95% confidence intervals.

The metastatic LNs were confined within the primary tumour region in 528 patients (17.6%) (Fig. 3). The most distant metastatic LNs were distributed outside the primary tumour region in 516 patients (17.2%): 0 < *D* ≤ 3 cm segment in 309 patients (10.3%), 3 < *D* ≤ 5 cm segment in 130 patients (4.3%), 5 < *D* ≤ 7 cm segment in 39 patients (1.3%) and 7 < *D* ≤ 10 cm segment in 34 patients (1.1%). Only four patients (0.1%) had LN involvement within >10 cm of the primary tumour.

Four cases with pericolic LN involvement >10 cm from the primary tumour were reported from different institutions. All had extensive LN metastasis, defined as the involvement of ≥10 LNs and/or main LN involvement (Supplemental Table S2).

Although the anatomical distribution of positive LNs was associated with the T stage, only 2 (0.1%) of 1443 patients with T3 tumours and 2 (0.4%) of 449 patients with T4a tumours had positive pericolic nodes located >10 cm from the primary tumour (Fig. 3). Similarly, the incidence of patients with pericolic positive nodes located >5 cm from the primary tumour was <1% in T1 tumours. The anatomical distribution of positive LNs in the pericolic region did not differ significantly depending on the location of the primary tumour (Table 3).

**Table 3:** Anatomical distribution of metastatic lymph nodes according to the location of the primary tumour and primary feeding artery in 1116 patients with lymph node involvement.

Location of the primary tumour and primary feeding artery	Total number of patients (%)	No metastasis in pericolic LNs	Pericolic tumour spread (location of the most distant metastatic pericolic LN)							Tumour spread along supplying arteries^a^	
			Primary tumour region	0 < *D* ≤ 3	3 < *D* ≤ 5	5 < *D* ≤ 7	7 < *D* ≤ 10	*D* > 10	*P*-value	Incidence	*P*-value
Tumour location [categorization 1]											
Cecum	137 (12.3)	9 (6.6)	72 (52.6)	37 (27.0)	15 (10.9)	2 (1.5)	1 (0.7)	1 (0.7)	0.27^b^	37 (27.0)	0.0055^e^
Ascending colon	369 (33.1)	15 (4.1)	165 (44.7)	109 (29.5)	50 (13.6)	16 (4.3)	12 (3.3)	2 (0.5)		80 (21.7)	
Transverse colon	148 (13.3)	20 (13.5)	62 (41.9)	39 (26.4)	19 (12.8)	5 (3.4)	3 (2.0)	0		53 (35.8)	
Descending colon	78 (7.0)	3 (3.8)	36 (46.2)	28 (35.9)	8 (10.3)	1 (1.3)	2 (2.6)	0		13 (16.7)	
Sigmoid colon	384 (34.4)	25 (6.5)	193 (50.3)	96 (25.0)	38 (9.9)	15 (3.9)	16 (4.2)	1 (0.3)		101 (26.3)	
Tumour location [categorization 2]											
Non-flexure site	974 (87.3)	53 (5.4)	470 (48.3)	272 (27.9)	108 (11.1)	36 (3.7)	32 (3.3)	3 (0.3)	0.19^c^	242 (24.8)	0.00062^e^
Hepatic flexure	91 (8.2)	18 (19.8)	38 (41.8)	19 (20.9)	13 (14.3)	0	2 (2.2)	1 (1.1)		36 (39.6)	
Splenic flexure	51 (4.6)	1 (2.0)	20 (39.2)	18 (35.3)	9 (17.6)	3 (5.9)	0	0		6 (11.8)	
Location of the primary feeding artery											
Primary tumour region	709 (63.5)	48 (6.8)	322 (45.4)	209 (29.5)	79 (11.1)	23 (3.2)	25 (3.5)	3 (0.4)	0.31^d^	199 (28.1)	<0.0001^f^
≤5 cm from the primary tumour	377 (33.8)	23 (6.1)	195 (51.7)	89 (23.6)	46 (12.2)	16 (4.2)	7 (1.9)	1 (0.3)		82 (21.8)	
5–10 cm from the primary tumour	28 (2.5)	1 (3.6)	10 (35.7)	11 (39.3)	4 (14.3)	0	2 (7.1)	0		3 (10.7)	
>10 cm from the primary tumour	2 (0.2)	0	1 (50.0)	0	1 (50.0)	0	0	0		0	

*D*: distance from the closest primary tumour edge (cm), LN: lymph node.

a Metastasis in intermediate and/or main LNs.

b *P*-value obtained by chi-square test is shown for 1044 patients with positive pericolic LNs, in which groups of ‘5< *D* ≤ 7’, ‘7< *D* ≤ 10’ and ‘*D* > 10’ are combined.

c *P*-value obtained by chi-square test is shown for 1044 patients with positive pericolic LNs, in which groups of ‘5 < *D* ≤ 7’, ‘7 < *D* ≤ 10’ and ‘*D* > 10’, and groups ‘hepatic flexure’ and ‘splenic flexure’ are combined, respectively.

d Test of Spearman's rank correlation coefficient.

e Chi-square test.

f Cochran-Armitage trend test.

### Anatomical distribution of the feeding arteries and their association with the location of the metastatic LNs

Among 2996 patients, a feeding artery flowed into the pericolic region within 10 cm of the primary tumour in 2989 (99.8%), and 1828 patients (61.0%) had a feeding artery that flowed into the primary tumour region (Fig. 2). In four of the seven cases with no feeding artery within 10 cm of the primary tumour, the primary tumour was located at the splenic flexure (Supplemental Table S3).

The distance of the primary feeding artery from the primary tumour differed significantly depending on the location of the primary tumour (Table 4). The feeding artery distributed most closely to the primary tumour in patients with cecum cancer, followed by those with sigmoid colon cancer. In patients with ascending, transverse or descending colon cancers, the primary feeding artery was more distant from the primary tumour as compared with in those with cecum or sigmoid colon cancers. In patients with tumours located at the splenic flexure, the proportion of patients with a feeding artery >5 cm from the primary tumour was higher, and the mean distance of the primary feeding artery from the primary tumour was greater than that in patients with tumours located at other colon segments.

**Table 4:** Location of the primary feeding artery according to the location of the primary tumour.

Primary tumour location		Total number of patients (%)	Location of the distal end of the primary feeding artery, number (%)			*P*-value^a^	Distance between the primary feeding artery and the primary tumour, mean (SD)^b^	*P*-value^c^
			Primary tumour region	≤5 cm from the primary tumour	>5 cm from the primary tumour			
Categorisation 1	Cecum	368 (12.3)	310 (84.2)	58 (15.8)	0 (0.0)	<0.0001	2.0 (0.9) cm	<0.0001
	Ascending colon	989 (33.0)	541 (54.7)	417 (42.2)	31 (3.1)		3.2 (1.8) cm	
	Transverse colon	459 (15.3)	231 (50.3)	196 (42.7)	32 (7.0)		3.6 (2.4) cm	
	Descending colon	215 (7.2)	123 (57.2)	76 (35.3)	16 (7.4)		3.8 (2.2) cm	
	Sigmoid colon	965 (32.2)	623 (64.6)	326 (33.8)	16 (1.7)		2.8 (1.7) cm	
Categorisation 2	Non-flexure site	2580 (86.1)	1626 (63.0)	895 (34.7)	59 (2.3)		3.0 (1.8) cm	
	Hepatic flexure	262 (8.7)	131 (50.0)	115 (43.9)	16 (6.1)	<0.0001^d^	3.4 (2.2) cm	0.081^d^
	Splenic flexure	154 (5.1)	71 (46.1)	63 (40.9)	20 (13.0)	<0.0001^e^	4.1 (2.9) cm	0.0011^e^

SD: standard deviation.

a Chi-square test and Fisher's exact test.

b In patients in whom the primary feeding artery is distributed outside the primary tumour region (n = 1168).

c ANOVA and t-test.

d *P*-values are shown for the difference between the ‘Non-flexure site’ and ‘Hepatic flexure” groups.

e *P*-values are shown for the difference between the ‘Non-flexure site’ and ‘Splenic flexure’ groups.

Despite a significant difference in the distribution of the primary feeding artery according to the location of the primary tumour, the anatomical distribution of the positive pericolic LNs was not significantly affected by the location of the primary feeding artery (Table 3). In contrast, metastasis in intermediate and/or main LNs was observed significantly more frequently in patients whose feeding artery was distributed within the primary tumour as compared with those in which it was more distant from the primary tumour (Table 3).

### Accuracy of intraoperative identification of the feeding arteries

Postoperative examination of the resected surgical specimens showed that the intraoperative marking for the feeding arteries was judged as correct in 2853 of the 2996 patients (95.2%). The accuracy of the intraoperative identification of the feeding arteries was significantly associated with BMI (*P* < 0.0001) (Table 5).

**Table 5:** Accuracy of intraoperative identification of the feeding artery.

Parameters	Categories	Intraoperative identification of the anatomical distribution of the feeding artery		*P*-value
		Accurate	Inaccurate	
Sex, number (%)	Male	1442 (94.4)	86 (5.6)	0.031^a^
	Female	1411 (96.1)	57 (3.9)	
Age, mean (SD)		68.8 (11.2)	67.5 (11.4)	0.16^b^
BMI, mean (SD)		22.5 (3.5)	23.9 (3.6)	<0.0001^b^
BMI, number (%)	<20	655 (97.2)	19 (2.8)	
	20 ≤ BMI < 25	1561 (95.8)	69 (4.2)	
	25 ≤ BMI < 30	568 (92.8)	44 (7.2)	
	≥30	69 (86.3)	11 (13.8)	
Intraoperative detection, number (%)	Under direct vision	2760 (95.1)	143 (4.9)	0.022^c^
	Laparoscopically	93 (100)	0 (0.0)	
Tumour location [categorisation 1], number (%)	Cecum	360 (97.8)	8 (2.2)	0.12^d^
	Ascending colon	938 (94.8)	51 (5.2)	
	Transverse colon	432 (94.1)	27 (5.9)	
	Descending colon	203 (94.4)	12 (5.6)	
	Sigmoid colon	920 (95.3)	45 (4.7)	
Tumour location [categorisation 2], number (%)	Hepatic flexure	242 (92.4)	20 (7.6)	0.071^d^
	Splenic flexure	148 (96.1)	6 (3.9)	
	Others	2463 (95.5)	117 (4.5)	

BMI: Body mass index, SD: standard deviation.

a Chi-square test with Yates' continuity correction.

b t-test.

c Fisher's exact test.

d Chi-square test.

### Postoperative oncological events according to pericolic LN status

None of the included patients experienced recurrence in the pericolic LN, although we did observe anastomotic recurrence, likely due to tumour implantation at the anastomotic site, in six patients (0.2%) during the observation period. The recurrence-free survival at 3 years was calculated according to the location of the most distant metastatic pericolic node as 80.4%, 79.1%, 76.2%, 62.7%, and 69.7% for patients with the most distant metastatic pericolic node within the primary tumour, within 3 cm of the primary tumour, within 3–5 cm, within 5–7 cm, and within 7–10 cm from the primary tumour, respectively (Supplemental Fig. S5). Of the four patents with distant pericolic nodal metastasis located >10 cm from the primary tumour, three developed recurrence in distant organs or non-mesenteric LNs postoperatively (Supplemental Table S2).

## Discussion

This is the first multicenter cohort study that prospectively assessed LN distribution in terms of the distance from the primary tumor and the anatomical association with feeding artery location according to the preplanned protocol with *in vivo* measurement. Based on the actual anatomical distribution of pericolic LN metastasis and its prognostic implication, we concluded that the 10 cm-rule is a valid and practical criterion to define ‘regional’ pericolic nodes.

The mean number of retrieved LNs was 30.8 per patient, which was much higher than figures reported by previous studies, which ranged from 11 to 14 in studies with large cohorts of stage II and III colorectal cancer patients.^11^, ^12^, ^13^, ^14^ This may have been due to the methodology adopted to retrieve the LNs in this study, that is not being used in clinical practice but allows us to provide most accurate and useful information for surgeons that can be used intraoperatively. In detail, surgeons, who are better trained in handling fresh specimens than pathologists, meticulously retrieved the LNs from unfixed surgical specimens. We are aware that these are not common practice in Europe but we believe that these achieved a satisfactory number of retrieved LNs in this study.^15^ Additionally, we assessed the anatomical distribution of the primary feeding artery and the paracolic nodes based on *in vivo* measurement of the bowel given that there is a remarkable difference in the length of colorectal segments between *in vivo* and *in vitro*. In deed, Goldstein et al.^16^ reported that bowel segments shrink by 40% of their original *in vivo* length following removal from the patient and after being left in an unfixed state for 10–20 min, and by up to 57% after fixation. In previous LN mapping studies,^17^^,^^18^ the distance of the pericolic nodes from the primary tumour was measured in resected surgical specimens, and no information was obtained regarding the association with the feeding artery location. We believe that the large number of pericolic nodes evaluated and the methodology used to assess their anatomical location ensured the quality of the LN mapping and clinical applicability of the results obtained in the present study.

Both the number of the pericolic nodes retrieved and the number of involved nodes decreased as the distance from the primary tumour increased. Notably, only approximately 1% or less metastatic foci were pathologically identified in pericolic nodes located >10 cm from the primary tumour, irrespective of the T-stage. Some previous studies in the late 1900s using the clearing technique reported cases of distant pericolic nodal metastasis, e.g., 37 cm from the primary tumour in Grinnel's study^19^ and 23 cm from the primary tumour in Morikawa et al.'s research^20^; however, no clear distinction between curative and palliative cases was made in these studies. In previous studies that only investigated patients who underwent curatively-intended surgery, the incidence of metastasis in pericolic nodes >10 cm from the primary tumour was rare, with 0% reported in Hida et al.'s study^21^ and 0.2% reported in Yamaoka et al.'s.^18^ In the present study, the estimated value of the incidence of pericolic lymphatic spread beyond 10 cm was 0.13%, and the upper limit of the 95% confidence interval remained low (0.26%). Moreover, we found no LN recurrence in the remnant pericolic region within our observation period of 44 months. In addition, all exceptional four cases with pericolic lymphatic spread beyond 10 cm had extensive mesenteric LN metastasis. Postoperatively, three of these patients developed recurrence in distant organs, indicating that such distant pericolic lymphatic spread is a form of systemic, rather than local disease. Based on these results, we concluded that pericolic LNs located within 10 cm of the primary tumour can be exclusively defined as ‘regional’, and should be resected as a routine surgical procedure. Current ASCRS guidelines recommend resection margins of 5–7 cm, but surgeons should be aware of the potential risk of pericolic nodal recurrence according to the resection margin; e.g., approximately 5% for a 5-cm margin and 2% for a 7-cm margin in patients with T4 tumor.

In Western countries, the length of bowel resection is thought to depend on the removal of the colon's arterial supply,^6^^,^^22^ and surgeons generally intend to resect the vascular arcade next to the primary feeding artery.^7^ Consequently, wide bowel resection is routinely employed in routine colon cancer surgery.^5^^,^^7^ In addition, due to the recent prevalence of CME, the length of the resected bowel is sometimes used as a standard to evaluate specimen quality, and wider resection of the bowel is regarded as a hallmark of high-quality colon cancer surgery.^23^ However, based on the results of the present study, surgical procedures that intend to extend bowel resection beyond the regional pericolic region do not contribute to improving the quality of surgery. There are well-ordered, orthodromic lymphatic frow from the pericolic lymphatic vessels to lymph nodes around feeding vessels, indicating the importance of understanding the area of pericolic area potentially harboring cancer cells with lymphatic spread in determining the optimal resection extent of mesentery. The concept of ‘regional’-LN-oriented bowel resection, not that of ‘the wider, the better’ is useful to achieve the optimal surgical procedure without excess or deficiency, and does not conflict with the concept of the CME technique. This is because the goal of CME is *en bloc* removal of the mesocolon harbouring metastatic LNs, with sharp anatomic dissection along the embryologic planes and preservation of the intact visceral fascia of the mesocolon,^24^ rather than extensive removal of the mesocolon.

In Japan, the 10-cm rule had been used as the criterion to classify pericolic nodes since the first edition of the Japanese staging manual for colorectal cancer (*General Rules for Clinical and Pathological Studies on Cancer of the Colon, Rectum and Anus*) was published in September 1977.^25^ Under this rule, pericolic nodes located within 10 cm of the closest edge of the primary tumour were regarded as regional LNs (Supplemental Fig. S2) and they were classified as either Group 1 (pericolic nodes ≤5 cm from the primary tumour) or Group 2 (pericolic nodes 5–10 cm from the primary tumour).^9^ As an exception, in patients with no feeding arteries distributing within 10 cm of the primary tumour, all LNs located between the primary tumour and the primary feeding artery were treated as regional nodes.^9^

In contrast, the *General Rules for Clinical and Pathological Studies on Cancer of the Colon, Rectum and Anus (7th edition)* published on March 2006^26^ adopted alternative criteria to define regional pericolic nodes—the feeding-artery-oriented rule (Supplemental Fig. S3), which is still used in Japan.^10^^,^^27^ This revision of the definition of regional pericolic node was based on theory concerning the estimated lymphatic flow from the pericolic towards the feeding artery region. Similarly, in the recent ASCRS guidelines, the grade of recommendation is ‘strong’ for the statement that the extent of resection of the colon should correspond to the lymphovascular drainage of the colon cancer site.^6^ However, there is no high-quality evidence to support its validity of feeding-artery-oriented bowel resection in clinical practice.

The present study shows that the distribution of the feeding artery differs depending on the location of the primary tumour. In general, the arcade of feeding arteries is wide in the transverse and descending colon, especially the splenic flexure site, as compared with the cecum and sigmoid colon.^19^ However, our results showed that the distribution of positive pericolic nodes did not differ according to the location of the primary tumour. Moreover, the location of the primary feeding artery did not significantly affect the anatomical distribution of positive pericolic nodes. These results indicate that the feeding-artery-oriented definition of regional pericolic nodes is not fully supported within a practical framework.

In addition, intraoperative identification of the feeding artery is not always accurate, especially in obese patients. According to our results, in over one in ten patients with a BMI ≥30, surgeons were unable to accurately identify the feeding artery location intraoperatively. This also indicates that the bowel resection policy based on the feeding-artery-oriented criteria for regional pericolic nodes may be suboptimal when standardising colon cancer surgery, and it may be even more so when it comes to the Caucasian European/American population with high BMI.

Unlike the situation in pericolic tumour spread, the location of the feeding artery impacted the incidence of central lymphatic spread along the supplying arteries. Specifically, incidences of tumour spread along the supplying arteries were significantly higher in patients with a primary feeding artery distributing in the pericolic region of the primary tumour. In addition, the incidence was lower in patients with a tumour located at the splenic flexure, where feeding arteries are sparsely located, than those with tumours at other sites. Namely, when there is a feeding artery in close proximity to the tumour, tumour cells may easily travel to the main LNs via the network of lymphatic vessels around the feeding artery, indicating the requirement for central vascular ligation achievement.

We identified seven exceptional cases with no feeding arteries located within 10 cm of the primary tumour. None of them had LN involvement in the pericolic nodes >10 cm from the primary tumour, intermediate LNs or main LNs. These results suggest that segmental resection of the colon with a 10-cm resection margin (D1 resection^10^^,^^27^) is as sufficient as curatively-intended surgery for such cases. However, as of now, considering the limited number of cases analyzed and the potential inaccuracy of intraoperative identification of the feeding artery, the conventional 10-cm rule^9^^,^^25^—the regional area extends to include pericolic LNs at the periphery of the primary feeding artery in cases having no feeding artery within 10 cm of the primary tumor—is more realistic from a practical point of view.

In conclusion, the distribution of the feeding artery did not impact the status of pericolic lymphatic spread, although it might affect that of central lymphatic spread along the supplying arteries that independently impairs survival outcomes.^28^ According to the actual anatomical distribution of pericolic LN metastasis and its prognostic implication, the 10-cm rule is a valid and practical criterion to define the ‘regional’ pericolic region, and this should be fully considered when determining the bowel resection margin. Our study has limitations. First, we did not examine micro-metastasis in the pericolic LN based on serial sectioning^29^ or immunohistochemical staining.^30^ However, given that we observed no LN recurrence in the remnant pericolic region even in patients with segmental resection of the colon with a 10-cm resection margin, micro-metastasis of clinical significance outside a 10-cm resection margin would be highly unlikely. Second, our findings are based on a Japanese population only. We are currently conducting an international prospective observational cohort study for optimal bowel resection extent and central radicality for colon cancer (T-REX study), which involves multiple institutions in seven countries (ClincalTrials.gov NCT02938481) and will allow us to determine whether the above findings are ubiquitously observed and can be generalised in patients with colon cancer in other countries.^31^

## Contributors

H.U., K.H., and K.S. devised the initial concept and design of the study. A.S., M.S., Ma.I., H.U., T.S., Y.H., T.K., Yus.K., H.I., K.M., K.Y., Ko.K,. K.T., Yuk.K., H.O., M.O., T.M., Ya.T., A.I., J.W., Yu.T., H.S., Ke.K., Y.A., and Mi.I. were responsible for data curation and verification of the raw data. T.N. analysed the data. All authours were involved in data interpretation and validation. H.U. wrote the original draft of the paper and produced the figures and tables of the manuscript. K.S., K.H., A.S., M.S., Ma.I., T.S., Y.H., T.K., Yus.K., H.I., K.M., K.Y., Ko.K., K.T., Yuk.K., H.O., M.O., T.M., Ya.T., A.I., J.W., Yu.T., H.S., Ke.K., Y.A., Mi.I. and T.N. were involved in reviewing and editing drafts of the paper and approving the manuscript. H.U. was involved in the project administration and KS played a role in the funding acquisition, supervision, and decision to submit the manuscript for publication.

## Data sharing statement

The data contains personal information, and the study participants have not consented to public data sharing. Data access requires permission from the Japanese Society for Cancer of the Colon and Rectum.

## Declaration of interests

All authors declare no competing interests.
