# Supplementary material for: Optimal bowel resection margin in colon cancer surgery: prospective multicentre cohort study with lymph node and feeding artery mapping
Source: Lancet Reg Health West Pac. 2023 Jan 18;33:100680. doi: 10.1016/j.lanwpc.2022.100680 (PMC10166781; doi:10.1016/j.lanwpc.2022.100680)
Supplement: Supplemental Figures S1–S5 and Tables S1–S3 [file mmc1.pdf]

Supplementary information

Table of contents

Supplementary Figures

Supplemental Figure 1	CONSORT diagram of the Prospective Multicentre Cohort Study for the Optimal Length of Bowel Resection in Colon Cancer Surgery (UMIN000030331)	Page 1
Supplemental Figure 2	The 10 cm-rule for defining regional pericolic nodes in the Japanese Classification of Colorectal Carcinoma (ref. 9, 25)	Page 2
Supplemental Figure 3	Feeding artery-oriented rule for regional pericolic nodes in the Japanese Classification of Colorectal Carcinoma (ref. 10, 26, 27)	Page 3
Supplemental Figure 4	Lymph node grouping for colon cancer in the Japanese Classification of Colorectal Carcinoma (ref. 9, 10, 25, 27)	Page 4
Supplemental Figure 5	Recurrence-free survival according to the location of the most distant metastatic pericolic node	Page 5

Supplementary Tables

Supplemental Table 1	Institutions that participated in the Prospective Multicentre Cohort Study for the Optimal Length of Bowel Resection in Colon Cancer Surgery	Page 6
Supplemental Table 2	Clinicopathological characteristics of the four patients with metastasis in pericolic lymph nodes located > 10 cm from the primary tumour	Page 7
Supplemental Table 3	Clinicopathological background of patients with no first feeding artery within 10 cm of the primary tumour	Page 8

Ueno H, et al. Optimal Bowel Resection Margin in Colon Cancer Surgery: Prospective Multicentre Cohort Study with Lymph Node and Feeding Artery Mapping

Supplemental Figure 1.

CONSORT diagram of the Prospective Multicentre Cohort Study for the Optimal Length of Bowel Resection in Colon Cancer Surgery (UMIN000030331)

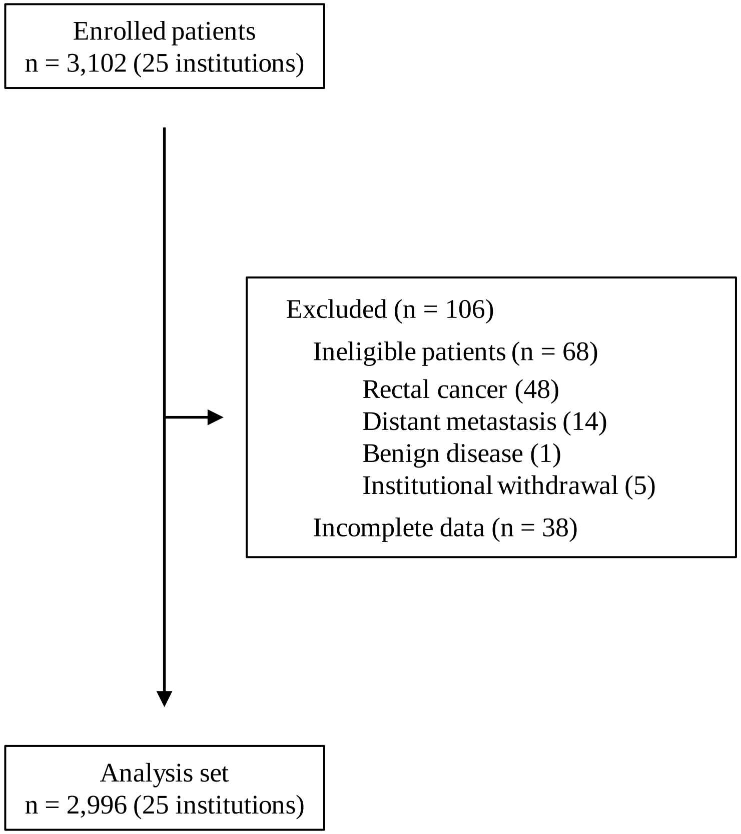

Supplemental Figure 2.

The 10 cm-rule for defining regional pericolic nodes in the Japanese Classification of Colorectal Carcinoma (ref. 9, 25)

Pericolic LNs located within 10 cm of the primary tumour on both sides are defined as regional nodes (A).
When there is no FA within 10 cm of the primary tumour, the regional area extends to include pericolic LNs at the periphery of the primary FA (B).

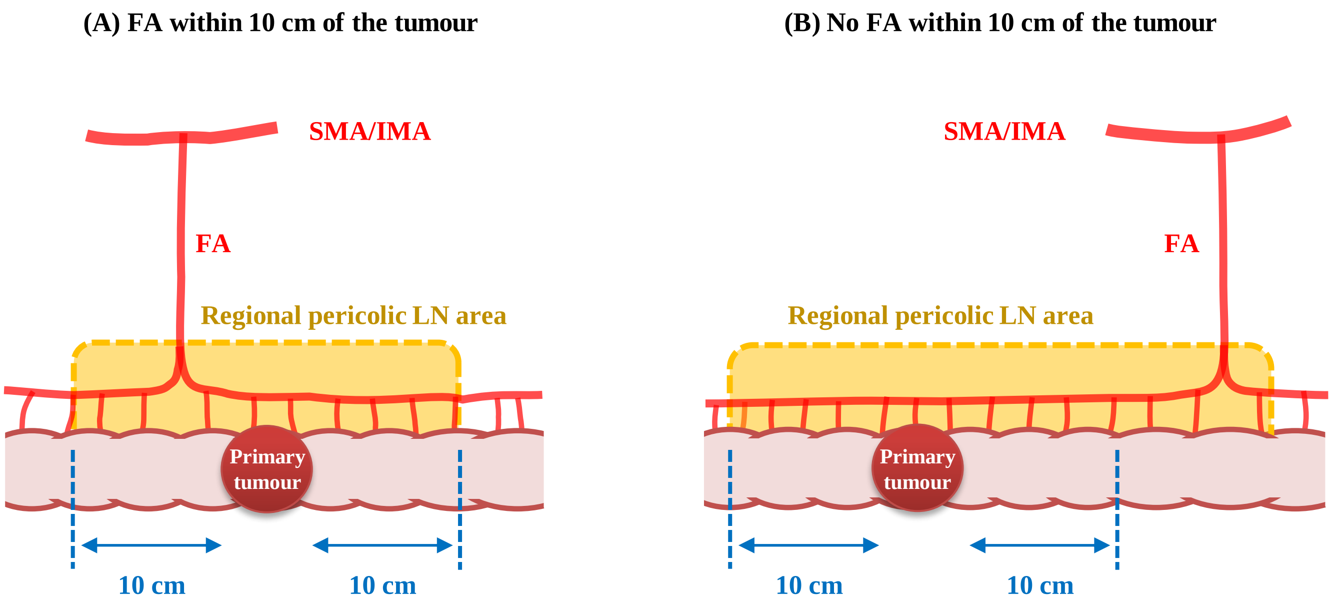

LN: Lymph node, FA: Feeding artery, SMA: Superior mesenteric artery, IMA, Inferior mesenteric artery Yellow: Regional pericolic LN area

Supplemental Figure 3.

Feeding artery-oriented rule for regional pericolic nodes in the Japanese Classification of Colorectal Carcinoma (ref. 10, 26, 27)

Regional pericolic nodes on both sides of the primary tumour where the primary FA enters the tumour area (A) and those on the opposite side of the primary FA entering outside of the tumour area (B) are defined by the 10-cm rule. Regarding the pericolic LNs located on same side of the primary FA, LNs between the primary tumour and distant 5 cm from the primary FA are defined as regional (B). When the vascular arcade next to the primary FA enters within 10 cm of the primary tumour, it is treated as the primary FA (C). When there is no FA within 10 cm of the primary tumour, the artery closest to the tumour is regarded as its FA (D).

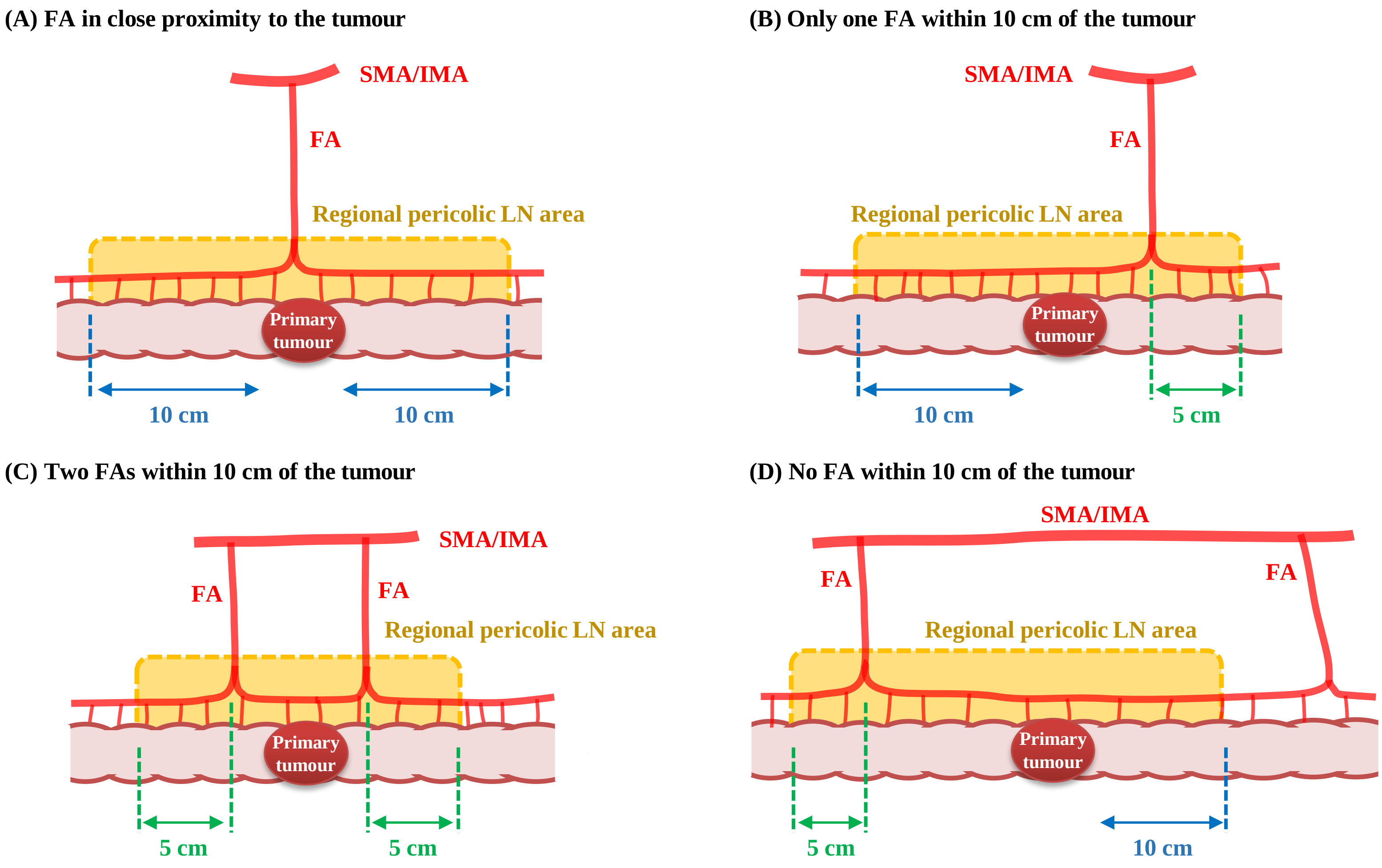

LN: Lymph node, FA: Feeding artery, SMA: Superior mesenteric artery, IMA, Inferior mesenteric artery Yellow: Regional pericolic LN area

Supplemental Figure 4.

Lymph node grouping for colon cancer in the Japanese Classification of Colorectal Carcinoma (ref. 9, 10, 25, 27)

Pericolic LNs: LNs along the marginal arteries and vasa recta of the colon

Intermediate LNs:

- Right-sided colon: LNs along the colic arteries
- Left-sided colon: 1) LNs along the left colic and sigmoid arteries; 2) LNs along the inferior mesenteric artery between the origin of the left colic artery and the origin of the terminal sigmoid artery

Main LNs:

- Right-sided colon: LNs at the origin of each colic artery
- Left-sided colon: LNs along the inferior mesenteric artery proximal to the origin of the left colic artery

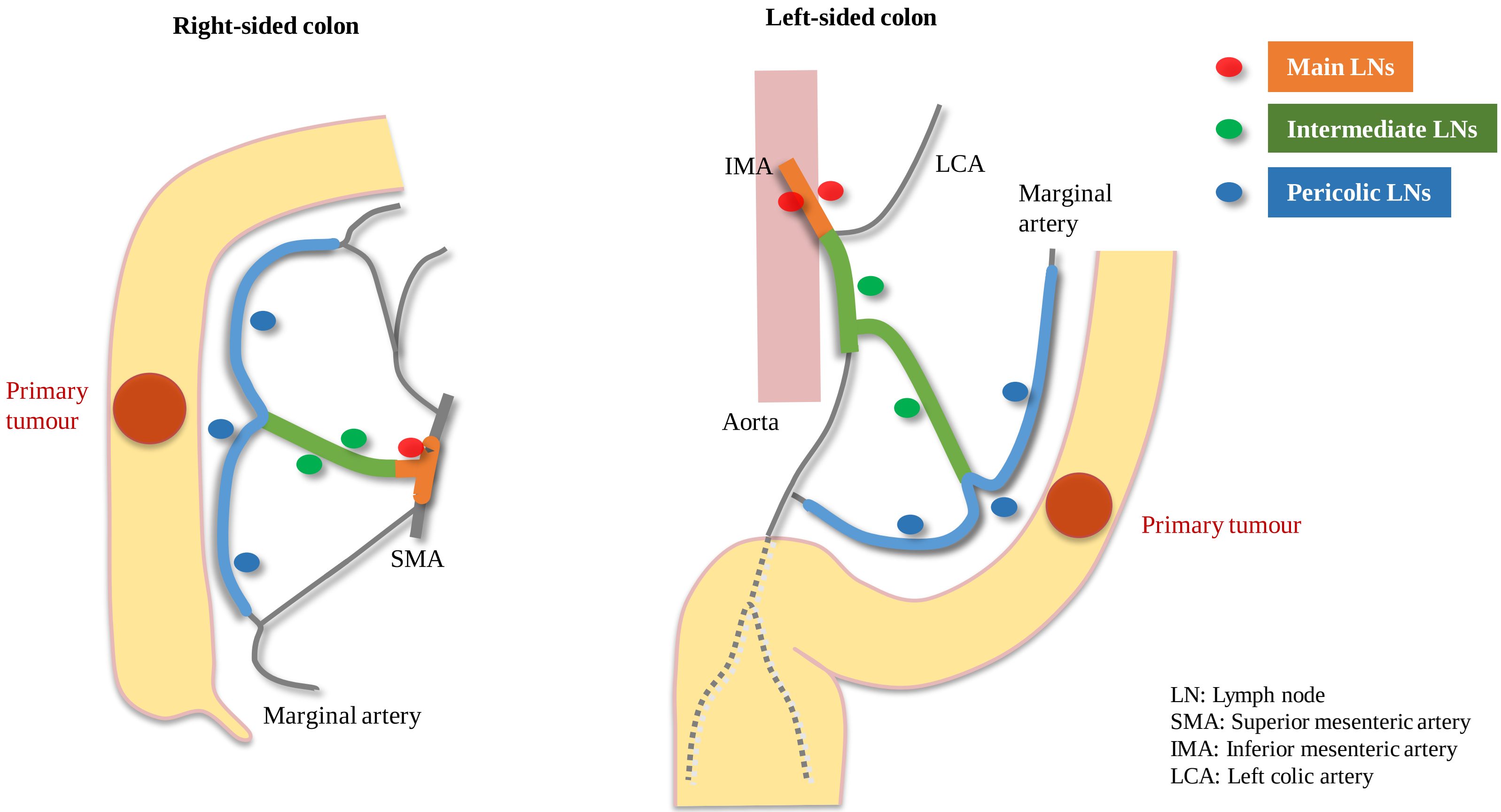

LN: Lymph node
SMA: Superior mesenteric artery
IMA: Inferior mesenteric artery
LCA: Left colic artery

Supplementary Figure 5.

Recurrence-free survival according to the location of the most distant metastatic pericolic node

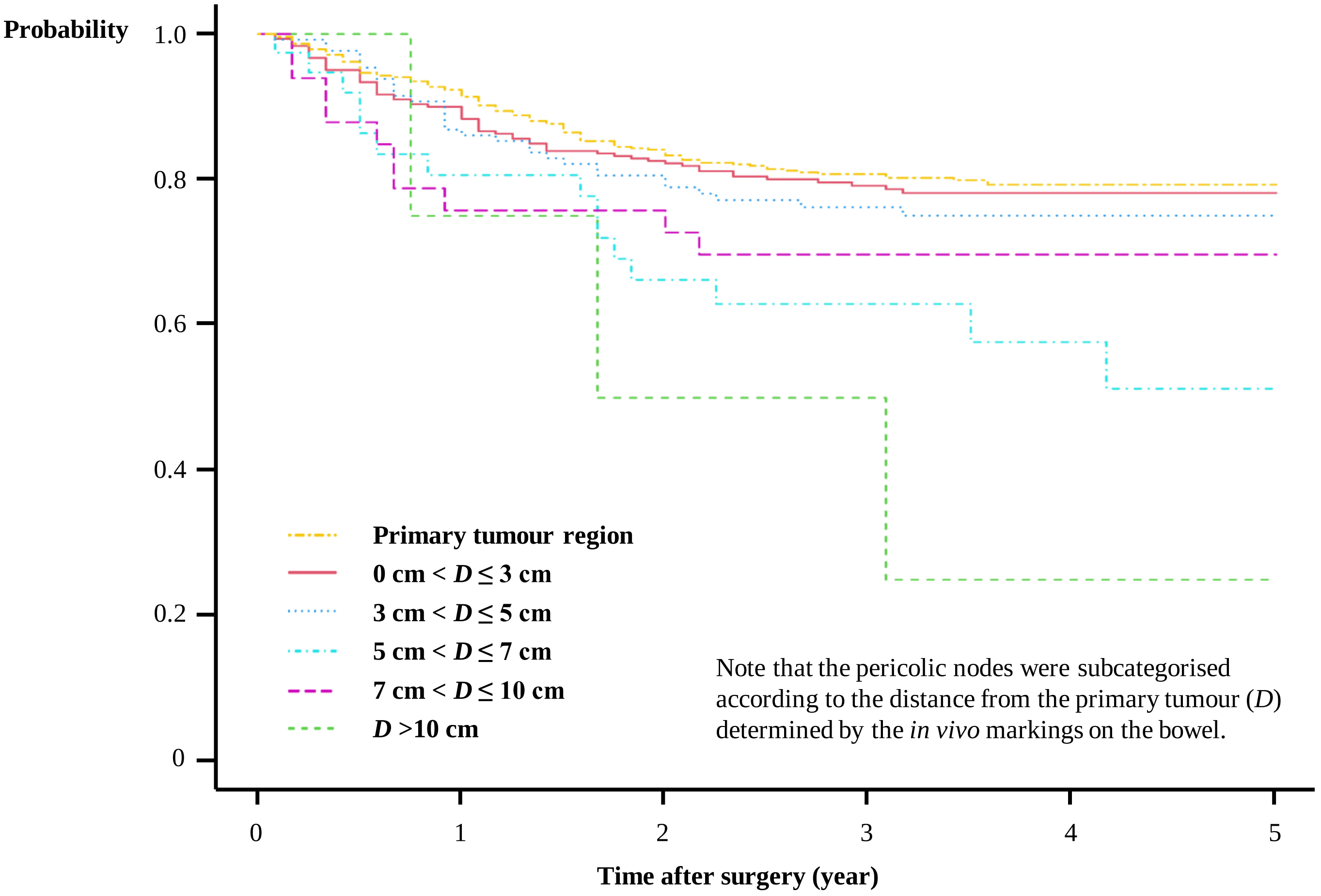

Number at risk

Primary tumour region	528	474	424	318	206	99
0 cm < $D \leq$ 3 cm	309	267	241	168	109	48
3 cm < $D \leq$ 5 cm	130	112	99	75	46	24
5 cm < $D \leq$ 7 cm	39	28	23	14	9	2
7 cm < $D \leq$ 10 cm	34	25	25	20	13	10
D >10 cm	4	3	2	2	1	1

Supplemental Table 1

Institutions that participated in the Prospective Multicentre Cohort Study for the Optimal Length of Bowel Resection in Colon Cancer Surgery

1.	Shizuoka Cancer Centre Hospital
2.	Kanagawa Cancer Centre Hospital
3.	National Cancer Centre Hospital East
4.	National Defense Medical College
5.	Yamagata Prefectural Central Hospital
6.	Teikyo University School of Medicine
7.	Keiyukai Sapporo Hospital
8.	Tokyo Medical and Dental University
9.	Saisei-kai Yokohama-shi Nanbu Hospital
10.	Wakayama Medical University
11.	Takano Hospital
12.	Aichi Cancer Centre Hospital
13.	Tokyo Metropolitan Cancer and Infectious Disease Centre Komagome Hospital
14.	National Cancer Centre Central Hospital
15.	Tochigi Cancer Centre
16.	Osaka International Cancer Institute
17.	Kyorin University School of Medicine
18.	Niigata Cancer Centre Hospital
19.	Yokohama City University
20.	Yokohama City University Medical Centre
21.	Mie University Graduate School of Medicine
22.	Shiga University of Medical Centre
23.	Teikyo University Chiba Medical Centre
24.	Kurume University School of Medicine
25.	Tokyo Women’s Medical University

Supplemental Table 2

Clinicopathological characteristics of the four patients with metastasis in pericolic lymph nodes located > 10 cm from the primary tumour

Case	Sex (age)	Tumour location	Tumour grade	T stage	Location of primary feeding artery	No. of harvested LNs	No. of involved LNs			Recurrence (organs)
							Total	Intermediate LN	Main LN	
1	Male (45)	C	G2	T4a	Primary tumour region	18	7	3	1	Yes (liver, peritoneum)
2	Male (73)	A[a)]	G3	T4a	Primary tumour region	27	5	0	1	No[d)]
3	Male (75)	S	G1	T3	1 cm distal to the primary tumour[c)]	26	10	3	0	Yes (liver, lung, non-mesenteric LNs)
4	Female (77)	A[b)]	G2	T3	Primary tumour region	25	11	2	2	Yes (non-mesenteric LNs)

C: Cecum, A: Ascending colon, S: Sigmoid colon, LN: Lymph node.

[a)]Hepatic flexure; [b)]non-hepatic flexure; [c)]1 cm from the primary tumour; [d)]69-month follow-up period.

Supplemental Table 3

Clinicopathological background of patients with no first feeding artery within 10 cm of the primary tumour

Case	Sex (age)	Tumour location-1	Tumour Location-2	Tumour grade	T stage	Distance between the distal end of the primary FAs and the primary tumour (cm)	No. of harvested LNs	No. of involved LNs		
								Pericolic	Intermediate	Main
1	Female (66)	A	Non-flexure	G2	T2	10.5	25	1*	0	0
2	Male (61)	T	Non-flexure	G2	T3	12.0	17	0	0	0
3	Male (77)	T	Splenic flexure	G1	T1	11.0	19	0	0	0
4	Female (78)	T	Splenic flexure	G1	T1	12.0	25	0	0	0
5	Female (67)	T	Splenic flexure	G1	T4a	13.0	26	0	0	0
6	Male (73)	D	Splenic flexure	G1	T1	12.0	19	1**	0	0
7	Male (87)	S	Non-flexure	G2	T2	13.0	16	0	0	0

A: Ascending colon, T: Transverse colon, D: Descending colon, S: Sigmoid colon, FA: Feeding artery, LN: Lymph node

*Primary tumour region; ** 3–5 cm from the primary tumour
